# Bacterial nanocellulose/calcium alginate hydrogel for the treatment of burns

**DOI:** 10.1590/acb393324

**Published:** 2024-07-15

**Authors:** Lauriene Luiza de Souza Munhoz, Luiz Carlos Guillens, Beatriz Candido Alves, Maria Gabriela Oliveira Ferreira do Nascimento, Andréia Bagliotti Meneguin, Fernanda Mansano Carbinatto, Gabriela Arruda, Hernane da Silva Barud, Andrea de Aro, Laura de Roch Casagrande, Paulo Cesar Lock Silveira, Thiago Antônio Moretti Andrade, Glaucia Maria Tech dos Santos, Guilherme Ferreira Caetano

**Affiliations:** 1 Centro Universitário Herminio Ometto de Araras – Graduate Program in Biomedical Sciences, Araras (SP), Brazil.; 2 Universidade Estadual de São Paulo – School of Pharmaceutical Sciences, Araraquara (SP), Brasil.; 3 Universidade de Araraquara – BioPolymer and Biomaterial Laboratory, Araraquara (SP), Brazil.; 4 Universidade de São Paulo – Institute of Physics, São Carlos (SP), Brasil; 5 Universidade do Extremo Sul Catarinense – Graduate Program in Science of Health – Criciúma (SC), Brazil.; 6 Centro Universitário Herminio Ometto de Araras – Graduate Program of Orthodontics, Araras (SP), Brazil.; 7 Universidade de São Paulo – Ribeirão Preto Medical School, Ribeirão Preto (SP), Brazil.

**Keywords:** Alginates, Anti-Inflammatory Agents, Burns, Cellulose, Hydrogels

## Abstract

**Purpose:**

Bacterial cellulose (BC) has shown high capacity for the treatment of wounds and burns, providing a moisty environment. Calcium alginate can be associated with BC to create gels that aid in wound debridement and contribute to appropriate wound healing. This study is aimed at characterizing and evaluating the use of bacterial cellulose/alginate gel in skin burns in rats.

**Methods:**

Cellulose and cellulose/alginate gels were compared regarding the capacity of liquid absorption, moisture, viscosity, and potential cytotoxicity. The 2nd degree burns were produced using an aluminum metal plate (2.0cm) at 120ºC for 20s on the back of rats. The animals were divided into non-treated, CMC(Carboxymethylcellulose), Cellulose(CMC with bacterial cellulose), and Cellulose/alginate(CMC with bacterial cellulose and alginate). The animals received topical treatment 3 times/week. Biochemical (MPO, NAG and oxidative stress), histomorphometry and immunohistochemical assays (IL-1β IL-10 and VEGF) were conducted on the 14th, 21st, 28th, and 35th days.

**Results:**

Cellulose/Alginate gel showed higher absorption capacity and viscosity compared to Cellulose gel, with no cytotoxic effects. Cellulose/alginate presented lower MPO values, a higher percentage of IL-10, with greater and balanced oxidative stress profile.

**Conclusions:**

The use of cellulose/alginate gel reduced neutrophils and macrophage activation and showed greater anti-inflammatory response, which can contribute to healing chronic wounds and burns.

## Introduction

People affected by skin injuries from burns need to receive immediate assistance, since the injury is systemic, mainly involving the cardiorespiratory, the digestive and the immune systems^1^. Burn injuries, particularly those that reach large proportions, develop to injuries that are difficult to treat, since the absence of healthy skin integrity impairs the maintenance of the body’s physiological homeostasis and often do not reach adequate healing^2^^-^ 3. In addition, the injured site becomes susceptible to infectious conditions, since the inflammation becomes exacerbated and persistent^3^^-^ 4.

Given the difficulties to treat large burned areas, the engineering of biomaterials has gained great importance in medicine. Investment in biotechnology aims at studying the application of natural and synthetic polymers, and materials with different properties have been available^5^. Studies involving natural substances to accelerate healing have increased due to the long period of treatment required depending on the chronicity and associated pathologies, in addition to medical care high costs^6^^-^ 7. Among natural polymers, polysaccharides such as cellulose and alginate have been considered for use in medicine due to their high availability and biocompatibility.

Bacterial cellulose (BC), produced by Gluconacetobacter, is a natural renewable bionanomaterial^8^^-^ 9. It has shown high potential for wounds and burns healing, standing out for its appropriate physicochemical properties, nanotechnological facet and nanofibers organized in a three-dimensional network that provides mechanical properties, and porosity^10^. The use of BC membrane with nano silver particles for chronic wounds has been demonstrated. The modulation of the inflammatory phase and balanced oxidative stress were evidenced. In addition, BC is extremely hydrophilic, which is easy to associate in a hydrogel, provides a moist environment for the wound bed, a physiological advantage for the treatment of burns^11^^-^ 12.

Moreover, in order to create a viscous hydrogel, which would favor the healing of burns, alginate can be incorporated into BC^13^^-^ 14 ^-^ 15. Alginate can be incorporated into BC, as it offers versatility and the ability to form ionically cross-linked smooth hydrogels with no need of occlusion^16^. The BC and alginate ionically cross-linked hydrogel resemble the extracellular matrix and preserve wound moisture. The ion exchange between calcium in the biomaterial and sodium in the wound leads to the formation of a stable gel^17^.

Therefore, the study of bacterial cellulose gel incorporated into alginate may favor skin healing, particularly chronic wounds and burns, and does not require occlusive dressings^18^^-^ 19. The present work evaluates the absorption capacity, humidity and viscosity of the BC and alginate hydrogel; evaluates the in vivo inflammatory response through neutrophils and macrophages activity, IL-10 and IL1-β factors; oxidative stress markers; angiogenesis and tissue formation.

## Methods

Studies were conducted according to the standards established by the Arouca law and approved by the ethical principles in animal research by the Ethics Committee on Animal Use of the Herminio Ometto Foundation, under process number 053/2018.

### Bacterial cellulose hydrogel with alginate

The gels used in this study, also known as hydrogels, were obtained through the patent Process Number: BR 10 2019 021848 7, produced by BioPolymer and Biomaterial Laboratory (BioPolMat), University of Araraquara (UNIARA).

### Moisture absorption capacity

Cellulose hydrogel and cellulose with alginate hydrogel were placed separately in 9 cm diameter plates and stored in a biofreezer at -80 °C for 48 h. The hydrogels were lyophilized for 48 h at -54.1 °C and 0.042 bar pressure. Precisely weighed circular sections of lyophilized hydrogel (n=3) were placed in contact with a specific volume (10 mL) at 37 °C of simulated human exudate, composed of CaCl_2_ (2.5 mM/L) and NaCl (142 mM/L)^20^. After 24 h and 48 h, the excess water was removed from the samples and weighed. The liquid absorption capacity was expressed in percentage: Moisture absorption (%) = (Swollen mass/Dry mass) x100.

Moreover, the moisture of the hydrogels was evaluated using a moisture meter with an infrared heat source ID-200 (Mars Científica). An aliquot of the sample was applied to a previously tared support and the support plus sample set had the mass recorded. Then, the radiation was focused on the sample, and the final mass (constant weight) after the process was recorded again.

### Rheological analysis

The shear rate was determined considering 0 to 100 s-1 for the ascending curve and 100 to 0 s-1 for the descending curve, for 120 seconds each, at a temperature of 32 °C. Consistency and flow indices for the quantitative analysis of flow behavior were determined by the equation: τ = κ · γ η. Where, “τ” is the shear force, “γ” is the shear rate, “κ” is the consistency index and “η” is the flow index.

### Evaluation of cell viability

Around 20 mg samples of both lyophilized hydrogels were incubated in 1 mL of cell culture medium at 37 °C for 24 h. The culture medium after 24 h was collected for using in the cell viability assay, performing serial dilution, considering the highest concentration 20 mg/mL and the lowest dilution 1.75 mg/mL, following the same as previously done^10^. GM07492 strain (human fibroblast, at 1.0×10^4^ density cells) were plated in 96-well culture plates and cultured overnight with supplemented Dulbecco’s Modified Eagle Medium (DMEM) medium for cell adhesion. The culture medium was replaced with 100 µL of the collected medium in contact with both lyophilized hydrogels, performing the serial dilution mentioned above and incubated again for 24 h. The MTT (3-(4,5-Dimethylthiazol-2-yl)-2,5-Diphenyltetrazolium Bromide) protocol was performed^10^. The formazan crystals were homogenized and the optical density was obtained at a wavelength of 570 nm.

### In vivo – animal study

Studies were conducted according to the Ethics Committee on Animal Use of the Herminio Ometto Foundation, under process number 053/2018. Male Wistar rats (3 months old, 250 g) were anesthetized with an intraperitoneal injection of ketamine hydrochloride (30 mg/kg) and xylazine hydrochloride (10 mg/kg). Trichotomy was performed on the back of all animals followed by the burning protocol, applying an aluminum metal plate (2.0 cm in diameter) coupled to an electronic device that maintains a constant temperature of 120 ºC. This plate was placed on the skin of the animal’s back for 20 seconds to produce 2^nd^ degree burns, according to the standard protocol^21^. The animals received an analgesic for pain relief (dipyrone sodium 50 mg/Kg), and intraperitoneal injection of tramadol (5mg/kg) every 12 hours, for 72 hours.

Rats were randomly divided into groups (n = 24 each): burns did not receive topical treatment (Non-treated); burns treated with carboxymethylcellulose hydrogel (CMC); burns treated with CMC + bacterial cellulose hydrogel (Cellulose); burns treated with CMC + bacterial cellulose + alginate hydrogel (Cellulose/Alginate). Each treatment was applied 3 times a week, and the burns were not covered with occlusive dressings. The animals were euthanized, according to ethics guidance on the 14^th^, 21^st^, 28^th^ and 35^th^ days (n = 6) for further histological and biochemical studies.

### Myeloperoxidase (MPO) and N-acetyl glucosaminidase (NAG)

The samples were homogenized using Polytron Brinkmann Instruments, USA), as previously reported, and placed in a 96-well plate. 25 μL of 3, 3’, 5, 5’ - tetramethylbenzidine (Sigma, USA) and 100 μL of H2O2 were added for MPO evaluation, while for NAG, 22 μL of the substrate (p-nitrophenyl-N-acetyl-D-glycosaminide) (Sigma-Aldrich) diluted in 50 μL citrate/PBS (50 mM, pH 4.5) was placed in contact with samples. The plate was incubated at 37 °C for 60 min, and 50 μL of 0.2M glycine buffer (pH 10.4) was added. Both absorbances were measured at 450 nm.

### Oxidative stress profile

Briefly, nitric oxide (NO) was estimated based on the production of nitrite. The samples were incubated with Griess reagent at 25 °C, and the absorbance was measured at 540 nm^22^. DCFH–H_2_O_2_ production was monitored in samples incubated with DCFH-DA. The excitation and emission wavelengths of 488 nm and 525 nm, respectively, were considered for data. Membrane damage was investigated by the protein carbonyl content using 2,4-dinitrophenyl hydrazide. The concentration of incorporated 2,4-dinitrophenylhydrazide was expressed per mg of protein at 370 nm. To determine the total thiol groups (sulfhydryl to measure membrane integrity), DTNB; Ellman’s Reagent, 5,5’-Dithiobis-(2-Nitrobenzoic Acid) was used, which reduced the thiol groups creating yellow derivative (TNB), measured by spectrophotometry at 412 nm^23^^-^ 24.

Superoxide dismutase (SOD) activity was evaluated by determining the inhibition of adrenaline autoxidation at 480 nm and the glutathione peroxidase (GSH) activity was measured by monitoring the reduced de-Nicotinamide adenine dinucleotide 2-Phosphate oxidation at 340 nm^25^. Reduced glutathione levels were determined in tissue homogenates in the presence of 5,5-dithiobis (2-nitrobenzoic acid) (DTNB). Color development resulting from the reaction was expressed per milligram of protein at 412 nm^26^. Protein content was determined using the Folin phenol reagent (phosphomolybdic–phosphotungstic reagent).

### Histological study

Samples were fixed in 10% buffered formaldehyde solution, and followed the creatin of paraffin blocks histological routine. Slices of 5.0 µm were stained by Gomori’s trichrome, and histological images were acquired at 200 x magnification using a LEICA^®^ DM- 2000 optical microscope for blood vessel evaluation using ImageJ^27^^-^ 28.

### Immunohistochemistry

Using the slices of 5.0 µm from histological routine, Novolink^TM^ Polymer Detection System kit (RE7280-K - Leica Biosystems Newcastle Ltd: Newcastle Upon Tyne, UK) was used for immunohistochemistry markers. The sections were subjected to antigen retrieval in a pressure cooker with citrate buffer pH 6.0 for 40 minutes, followed by endogenous peroxidase and protein block, as recommended by the kit. The following primary antibodies were incubated overnight at 4 °C: IL-1β (1:350; sc-7884), IL-10 (1:200; sc-8438) and VEGF (1:200; sc-152) from Santa Cruz Biotechnology-USA. The slices were incubated for 30 min with Post Primary followed by 3, 3’ diaminobenzidine tetrahydrochloride (DAB) working solution for 5 min^27^.

### Data

Experimental data are represented as the mean ± standard error of the mean. Data were analyzed using the GraphPad Prism 8.0.2 software (GraphPad Software, San Diego, CA, USA). The Shapiro–Wilk normality test was conducted. One-way analysis of variance with Tukey post-hoc or Kruskal–Wallis with Dunn’s post-hoc tests were applied. Values of p < 0.05 show statistical evidence, with a 95% confidence interval. Significance levels were set at: *p < 0.05; **p < 0.01; ***p < 0.005; ****p < 0.0001.

## Results

### Moisture incorporation capacity (absorption) and rheological analysis

Table 1 presents the absorption values (in percentage) of simulated human exudate in two different periods (24 h and 48 h) using cellulose hydrogel and cellulose hydrogel associated with alginate. The cellulose hydrogel with alginate showed an absorption capacity approximately 25% greater than the hydrogel without alginate at both times of analysis. Table 2 presents the moisture values (in percentage) of both lyophilize hydrogels. Cellulose/alginate hydrogel showed a slightly higher moisture than cellulose hydrogel. Table 3 presents the apparent viscosity values of both hydrogels. The cellulose hydrogel with alginate showed higher viscosity, approximately 12% higher.

**Table 1:** Moisture incorporation capacity (absorption) of cellulose hydrogel and cellulose hydrogel with alginate.

Hydrogel sample	Moisture absorption capacity after 24 h (%)	Moisture absorption capacity after 48 h (%)
Cellulose	1365 ± 23	1490 ± 6
Cellulose/Alginate	1784 ± 17	1950 ± 20

**Table 2:** Moisture values for cellulose hydrogel and cellulose with alginate.

Hydrogel sample	Moisture (%)
Cellulose	95,7 ± 0,8
Cellulose/Alginate	98,2 ± 1,1

**Table 3:** Apparent viscosity values.

Hydrogel sample	Viscosity (Pa.s)
Cellulose	140,31
Cellulose/Alginate	157,6

### Cell viability

**Figure 1:**
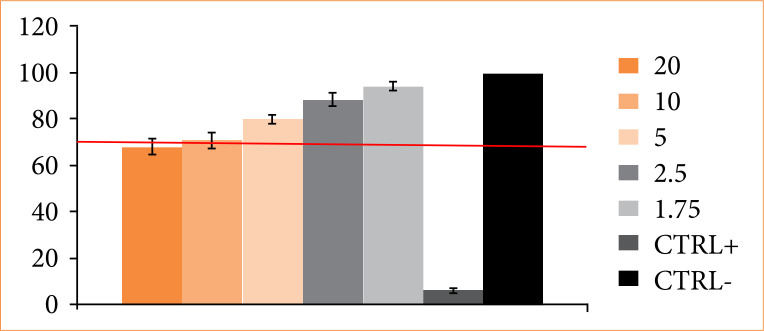
Evaluation of cell viability (%) considering cellulose hydrogel with alginate at different concentrations (1.75 to 20 mg). Human fibroblast line GM0749 and MTT assay were used. The red line corresponds to the recommended minimum viability for any samples not to be considered cytotoxic. CTRL- is the negative control (only medium), while CTRL+ is the positive for cytotoxicity.

Figure 1 presents the cell viability data in percentage relative to control with only medium, as 100%. All concentrations used (1.75 to 20 mg of lyophilized product / mL of culture medium) did not show toxicity at the cellular level, with viability greater than 70%. It showed a dose-dependent effect on cell viability. The more concentrated dose (20 mg) presented lower cell viability (around 70%).

### Inflammatory profile through evaluation of neutrophil activation by myeloperoxidase (MPO), macrophage activation by N-acetylglucosaminidase (NAG), IL-1β and IL-10

**Figure 2:**
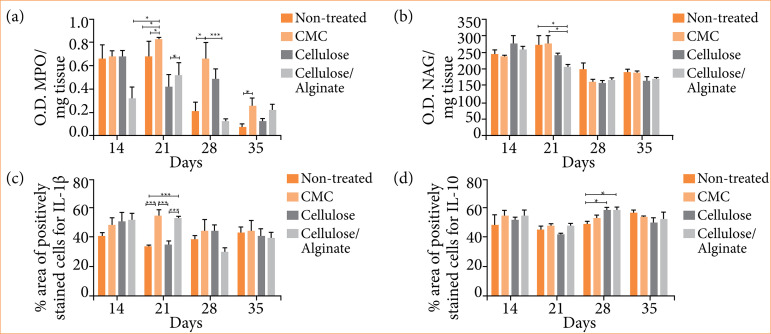
Valuation of inflammatory profile. a) MPO (myeloperoxidase) and b) NAG (N-acetylglucosaminidase) activity per mg of tissue by biochemical assay; percentage of area by immunohistochemistry of c) IL-1β and d) IL-10. Experimental groups: Non-treated, CMC, Cellulose and Cellulose/Alginate evaluated on the 14^th^, 21^st^, 28^th^ and 35^th^ days.

Figure 2a presents the results of MPO and NAG. On the 14th day, Cellulose/Alginate group presented lower MPO compared to the other three groups. On the 21^st^ day, it was observed that both Cellulose and Cellulose/Alginate groups presented lower MPO than CMC and Non-treated. On the 28^th^ day, Cellulose/Alginate and Non-treated groups showed lower MPO than CMC group, while on the 35th day, all four groups had a decrease in MPO, however, the CMC group still showed to have a higher MPO than the non-treated group. Figure 2b demonstrates all four groups presented similar NAG on the 1^4th^ day. However, on the 21st day, the Cellulose/Alginate group had lower enzymatic levels compared to the Non-treated and CMC groups. After 28 and 35 days, all groups presented similar NAG levels.

Figures 2c and 2d present IL-1β and IL-10 evaluations, respectively. Both Cellulose/Alginate and CMC groups presented higher IL-1β compared to Non-treated and Cellulose groups on the 21^st^ day. There was no significant data for the other experimental periods. A higher percentage of IL-10 in Cellulose and Cellulose/Alginate groups was observed when compared to Non-treated and CMC groups. In the other experimental periods, the four groups presented a similar percentage.

### Oxidative stress, oxidative damage and antioxidants markers

**Figure 3:**
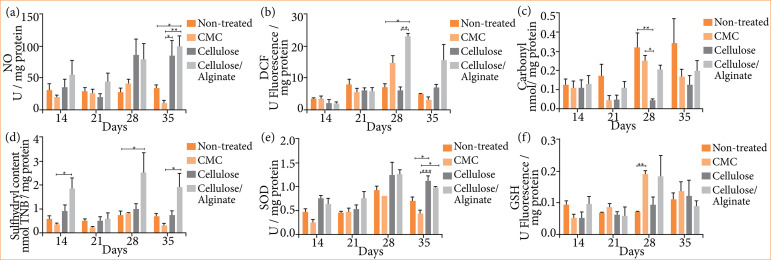
Oxidative stress profile. Dosage of a) NO, b) DCF, c) Carbonyl, d) Sulfhydryl, e) SOD, and f) GSH. Experimental groups: Non-treated, CMC, Cellulose and Cellulose/Alginate evaluated on the 14^th^, 21^st^, 28^th^ and 35^th^ days.

Relevant markers such as nitric oxide (NO), dichlorofluorescein diacetate (DCF), carbonyl and sulfhydryl were used. On fig.3a, on the 35th day, Cellulose/Alginate presented significantly different NO quantification when compared to the other groups. On fig. 3b, the quantification of DCF (indicative of the presence of H_2_O_2_) was higher and showed significance on day 21 in the group that received Cellulose/Alginate compared to the Non-treated and Cellulose groups.

Regarding the quantification of Carbonyl, the Non-treated group presented higher levels compared to the group that received only Cellulose, in addition to CMC group presenting higher levels than Cellulose, both on the 28^th^ day (Fig. 3c). Regarding the evaluation of sulfhydryl, it was observed that, on days 14^th^, 28^th^ and 35^th^, the group treated with Cellulose/Alginate presented higher levels. Specifically, on days 14 and 35, these levels were higher when compared to the group receiving CMC, and on day 21 they were higher compared to the non-treated group (Fig. 3d).

Regarding the evaluation of the antioxidant profile, Glutathione (GSH) and Superoxide Dismutase (SOD) were performed. It can be observed in Fig. 3e that there was statistical significance only on the 35^th^ day. At that point, the group that received Cellulose had higher levels of GSH compared to the non-treated group and the group that received CMC. There was also significance between the group that received Cellulose and the group that received CMC. With regard to the levels of Superoxide Dismutase (SOD), a significant increase was observed only on day 28, when compared the CMC and Non-treated groups (Fig. 3f).

### Tissue formation: angiogenesis

**Figure 4:**
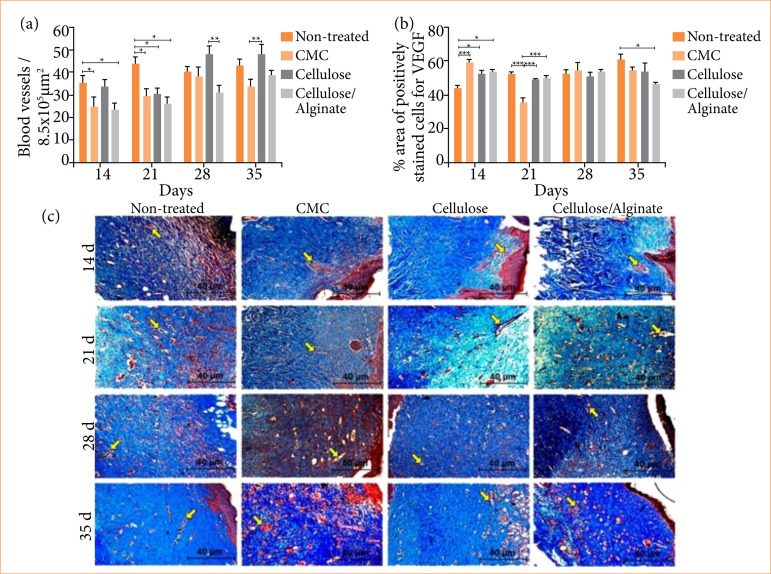
Evaluation of angiogenesis a) Quantification of the number of blood vessels. b) Percentage of area by immunohistochemistry of VEGF; c) Gomori’s trichrome staining images at 200x. Experimental groups: Non-treated, CMC, Cellulose and Cellulose/Alginate evaluated on the 14^th^, 21^st^, 28^th^ and 35^th^ days.

Figure 4b shows the quantification of the percentage area of Vascular Endothelial Growth Factor (VEGF) by immunohistochemistry. On the 14^th^ day, the CMC, Cellulose and Cellulose/Alginate groups presented a higher percentage in relation to the Non-treated group. On the 21^st^ day, the Non-treated, Cellulose and Cellulose/Alginate groups presented a higher percentage in relation to the CMC group, while on the 35^th^ day, the Cellulose/Alginate group presented a lower percentage in relation to the Non-treated Group.

In the graph shown in Fig. 4a, the quantification of blood vessels was performed through histomorphometry. It can be observed that the Non-treated group presented a higher number of blood vessels than the CMC and Cellulose/Alginate groups on the 14^th^ day and higher than the three groups on the 21^st^ day. On day 28, the Cellulose group presented a greater amount of blood vessels when compared to the Cellulose/Alginate group. And still on the 35^th^, the cellulose group was superior to the CMC group.

Figure 4c shows representative photomicrographs of each group throughout the experimental period by the histological protocol to the final 200x magnification stained by Gomori’s Trichrome (TG). The presence of the extracellular matrix is observed in blue and the blood vessels in red (yellow arrows).

### Tissue formation: connective tissue and assessment of tissue formation.

**Figure 5:**
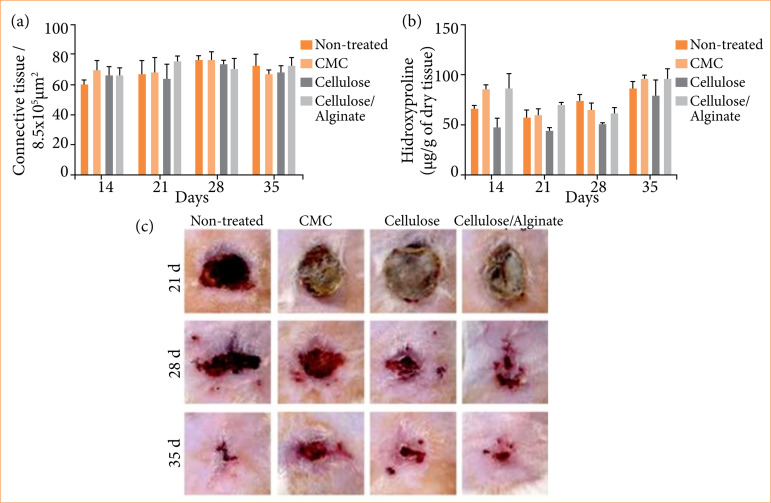
Connective tissue and assessment of tissue formation. a) Connective tissue; b) Hydroxyproline; c) Representative images of clinical evolution of the burn wounds. Experimental groups: Non-treated, CMC, Cellulose and Cellulose/Alginate evaluated on the 21^st^, 28^th^ and 35^th^ days of experiment.

Figure 5a presents the tissue formation measured by the percentage of connective tissue (% area). No significant difference was observed for connective tissue formation. Figure 5b shows the quantification of hydroxyproline in relation to collagen synthesis, which indicates scar progression. Regarding hydroxyproline, on the 14^th^ day and on the 21^st^ day, the CMC group presented a higher amount of collagen when compared to the cellulose group.

Figure 5c displays representative images of the animals’ burns after 21, 28, and 35 days of treatment. Days 7 and 14 were not considered due to the presence of a thick and resistant crust, which began to detach from the wounded area after day 14, as a consequence of the granulation tissue. Cellulose and Cellulose/Alginate groups seem to present enhanced re-epithelialization on the 28^th^ day. However, on the 35^th^ day the four treatments look similar.

## Discussion

Skin lesions are an important public health problem worldwide. Injuries that do not heal in the expected time can result in chronic conditions. Skin injury by burns is even critical once it evolves to a systemic effect. Faced with the need for alternative treatments and their applicability, bacterial cellulose has been studied for wound healing^29^^-^ 30. Bacterial cellulose-based biomaterials for tissue engineering have been considered due to its natural origin, biocompatibility, appropriate mechanical resistance, renewability, ease of acquisition, and skin application^31^^-^ 32.

The association of bacterial cellulose with alginate as gel (or hydrogel) is related to the preservation of wound moisture and the absorption of exudates, crucial factors for burn healing^11^^-^ 17. Chiaoprakobkij et al. (2019) reported the use of BC films incorporated with alginate, gelatin and glycerol. Although the authors mentioned advantages of using gelatin and glycerol, such as flexibility, water absorption capacity and oxygen permeability, products made only with BC and alginate showed appropriate mechanical properties (resistance and tensile strength), hydrophilicity and microenvironment for growth of keratinocytes and fibroblasts^33^.

Our results indicated that the addition of alginate on BC increased the gel viscosity and enhanced the liquid absorption (simulation of organic exudate), characteristics that are important to absorb wound secretions, and to maintain local humidity with no cytotoxicity. This demonstrates a cell viability in human fibroblast above 70% even at the highest concentrations. These findings prove that using cellulose with alginate in gel form could favor the wound healing which presented advantages comparing to only BC or occlusive dressings that tend to keep the wound bed with low humidity^34^.

The healing properties of the bacterial cellulose gel associated with calcium alginate were evaluated on Wistar rats with burn injuries. Neutrophils are the first cells to arrive at the injured site and play an important role in production of free radicals, which initially protect the injured bed, however, in excess, they become toxic to cells and tissue^35^. Exacerbated neutrophil activation could prolong the inflammatory phase, favoring collagen degradation or preventing its synthesis^28^^-^ 36. The use of Cellulose reduced the activation of neutrophils as from the 21^st^ day. Interestingly, Cellulose/Alginate gel advanced such effect to the 14^th^ day, positively modulating MPO during the inflammatory phase. The same was observed for macrophage activation by Cellulose/Alginate gel. Macrophages play an important role on tissue debridement, secretion of cytokines, interleukins, like IL-1β, and growth factors, which are vital in the inflammatory to tissue formation phase. Even though Cellulose/Alginate group presented high IL-1β on the 21st day, similar to CMC group, both different to Non-treated group, on the 28^th^ day Cellulose/Alginate showed reduced expression, but higher IL-10, an anti-inflammatory interleukin, corroborating with MPO and NAG data. IL-10 is a regulatory factor acting as suppressor of the inflammatory pathways, controlling the immunopathology caused by pro-inflammatory cytokines, triggering the physiological process of healing through the production of extracellular matrix (ECM), angiogenesis, cell proliferation and differentiation. Once the inflammatory phase is controlled, tissue formation is activated to replace the injured area. Cellulose/Alginate gel treatment is supposed to positively modulate the inflammatory phase, a key factor for chronic wounds. Moreover, the results showed a greater but balanced oxidative stress profile by using Cellulose/Alginate gel. Although NO and DCF presented higher data, SOD and GSH (antioxidant molecules), also higher, might be able to neutralize the effects of oxidants and prevent oxidative damage, which were present during the inflammatory phase and could contribute to avoiding the harmful effects.

Angiogenesis is related to the intense need for nutrition and oxygenation at the injured site, both during the inflammatory phase, where there is a large energy expenditure, and for tissue formation. The results did not provide evidence of angiogenesis after treatment with Cellulose/Alginate gel. Considering the MPO and NAG modulation discussed before, probably it was not required greater nutrition, which could explain the lack of evidence of angiogenesis, modulated via negative feedback. Although the Cellulose group presented high VEGF percentage, Non-treated group had similar results. The lower percentage of VEGF in Cellulose/Alginate group could indicate an expressive healing process in relation to the other groups. However, the evaluation of the connective tissue was similar among the groups. The expression of collagen I and III, as well as many other structural proteins, was not evaluated, but could indicate the matured stage of the new tissue.

Pandey et al. (2017) evaluated the use of a non-toxic and biodegradable vegetable cellulose hydrogel for skin burns in an animal model. The hydrogel proved to be highly porous and with adequate swelling^37^. The results also showed that hydrogel promoted faster burn healing, by re-epithelialization and fibroblast proliferation, which corroborate our findings. Kwak et al. (2015) and Muangman et al. (2011) investigated the effectiveness of bacterial cellulose membranes for burns. The authors noted that cellulose membranes could accelerate the healing process of burns without showing signs of irritation and/or skin allergy^38^^-^ 39. However, none of these studies evaluated collagen specifically.

The literature describes faster re-epithelialization and wound healing after treatment with cellulose and/or cellulose and alginate, which can be explained by the mediation of the inflammatory phase observed in this study. Although more evident, re-epithelialization was not observed according to the clinical follow-up of the burns, the results presented are in line with the literature. Clinically, burns are considered a systemic aggravation injury with difficult inflammation control. The results obtained demonstrate the potential use of cellulose and alginate gel for burn healing, which could provide even more advantages for the treatment of chronic wounds, particularly difficult to heal due to persistent inflammation.

## Conclusion

The incorporation of alginate into the bacterial cellulose gel increased the absorption property and the viscosity with no *in vitro* cytotoxicity. In addition, the gel as a treatment for burns *in vivo* regulated the activation of pro-inflammatory cells and anti-inflammatory cytokines, which may favor the healing of skin wounds.

## Data Availability

The data will be available upon request.
